# Case Report: A rare case of hemoptysis: multiple vascular variations

**DOI:** 10.3389/fmed.2025.1585686

**Published:** 2025-06-05

**Authors:** Beining Zhang, Jiangye Wang, Ninggang Zheng

**Affiliations:** Department of Interventional Oncology, Gansu Provincial Hospital, Lanzhou, Gansu, China

**Keywords:** hemoptysis, bronchial artery, vertebral artery, intercostal artery, bronchial artery embolization

## Abstract

Bronchial artery embolization (BAE) is an effective treatment for hemoptysis, with potential complications including pain and spinal ischemia. We report a rare case in which the right bronchial artery communicated with the right intercostal arteries, and the right intercostal artery had an anastomosis with the right vertebral artery. Additionally, the left bronchial artery was found to have a connection with the left vertebral artery.

## Introduction

Hemoptysis is the process of bleeding from the trachea, bronchi, or pulmonary tissue, which is expelled through coughing from the mouth. It is a common complication of respiratory system diseases (1). In recent years, with the advancement of vascular interventional techniques, bronchial artery embolization (BAE) has become the primary treatment for hemoptysis. Procedure-related complications may include pain, cerebral infarction, and spinal ischemia, among others (2, 3). This article reports a rare case of hemoptysis: multiple vascular variants.

## Case report

The patient is a 50-year-old woman with a history of intermittent cough and hemoptysis for 8 years, without any significant past medical history. She was admitted to the hospital due to worsening hemoptysis. Chest computed tomography (CT) revealed the following findings: a lamellar hyperdense shadow in the middle lobe of the right lung, suggestive of an hemoptysis reaction。.

**Figure 1:**
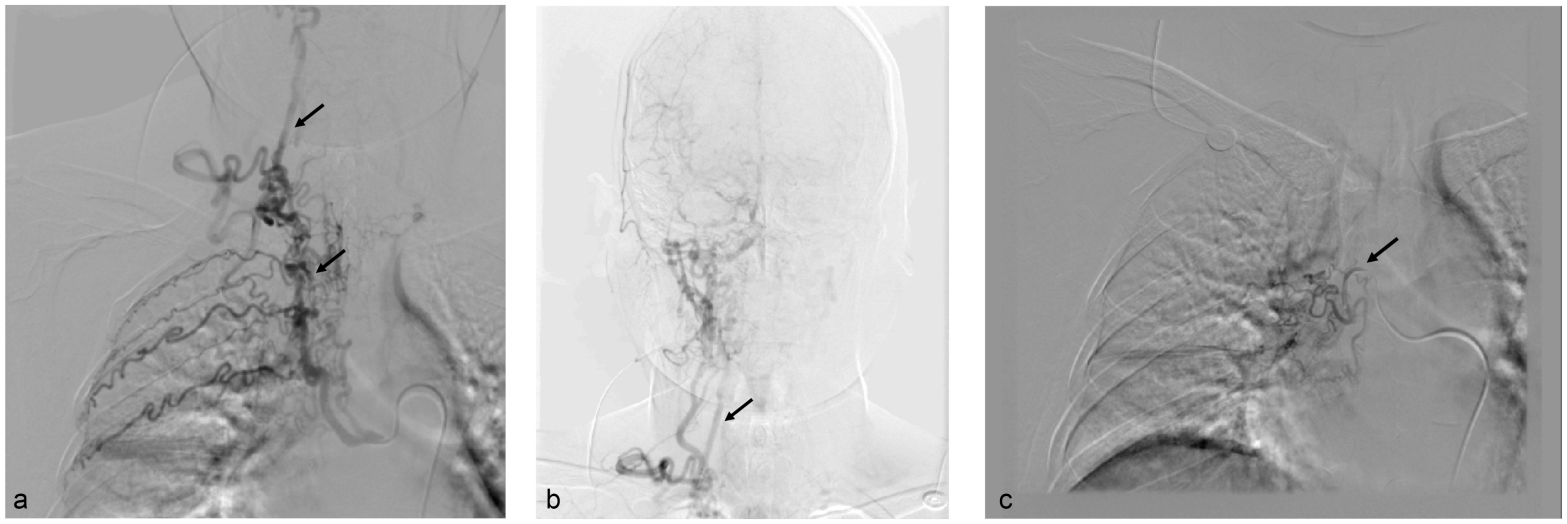
**(a,b)** Right bronchial artery angiography revealed an anastomosis between the intercostal arteries and the right bronchial artery, as well as communication between the intercostal arteries and the right vertebral artery. **(c)** Microcatheter angiography revealed the right bronchial artery.

**Figure 2:**
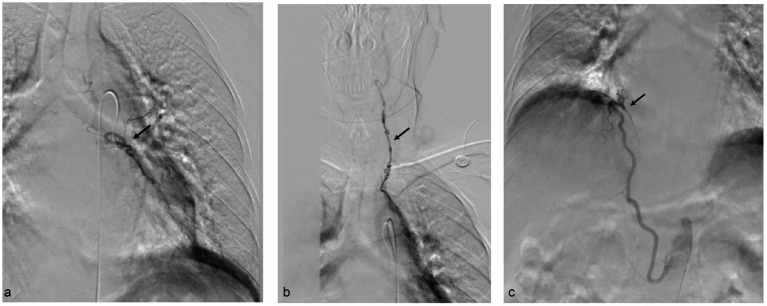
**(a)** Left bronchial artery. **(b)** The left bronchial artery was found to have an anastomosis with the left vertebral artery. **(c)** Angiography of the right phrenic artery revealed vascular abnormalities.

The patient underwent bronchial artery embolization. Right bronchial artery angiography revealed vascular abnormalities, with communication between the right bronchial artery and the first, second, and third right intercostal arteries. Additionally, the right intercostal artery was found to have an anastomosis with the right vertebral artery (Figures 1a,b). A microcatheter was carefully selected to access the non-communicating bronchial artery, followed by embolization with 500–700 μm and 700–900 μm embolic microspheres (Figure 1c). Left bronchial artery angiography revealed vascular lesions. The left bronchial artery was selectively catheterized using a microcatheter, followed by embolization with 700–900 μm embolic microspheres (Figure 2a). Further angiographic evaluation of another bronchial artery revealed an anastomosis with the left vertebral artery (Figure 2b). The right inferior phrenic artery also demonstrated pathological changes. Embolization was performed using 700–900 μm embolic microspheres, 1,000–1,400 μm gelatin sponge particles, and three miniature spring coils (Figure 2c). Postoperatively, the patient received continued hemostatic, anti-infection, and other symptomatic supportive treatments. No further episodes of hemoptysis occurred, and the patient was discharged in stable condition after recovery.

## Discussion

Hemoptysis is a common clinical emergency with a mortality rate exceeding 70%, posing a significant threat to patient safety (4). For patients with recurrent hemoptysis unresponsive to conservative treatment, surgical intervention is often required; however, such procedures can lead to a significant decline in the patient’s quality of life (5). Bronchial artery embolization (BAE) is a minimally invasive, effective, and repeatable therapeutic technique, and it has become the first-line treatment for hemoptysis (6).

Studies have shown that 90% of the blood supply to hemoptysis originates from the bronchial arteries, although other arteries, including the intercostal arteries, inferior phrenic arteries, and internal mammary arteries, also contribute to the vascular supply (2). The most serious complication associated with bronchial artery embolization is paraplegia/paralysis due to transient or permanent spinal cord injury (incidence 0.6–4.4%) (7). Other major complications include: (a) transient chest pain (with an incidence rate as high as 91%); (b) dysphagia (with an incidence rate of up to 18%); (c) spinal cord ischemia (approximately 6% incidence); (d) aortic or subintimal dissection of the bronchial arteries (also about 6% incidence). It is important to note that spinal cord injury, as a characteristic severe complication, is closely related to the reflux of embolic agents into critical vessels such as the spinal artery. In contrast, chest pain and dysphagia are often associated with localized ischemic edema following embolization (8, 9).

We observed communication between the right intercostal artery and the right bronchial artery. Additionally, the right intercostal artery was found to communicate with the right vertebral artery. The development of the intercostal arteries begins early in embryogenesis, shortly after the formation of the endocardial heart tube. The heart tube forms the atrial outflow tract, which consists of an aortic sac and six pairs of aortic arch arteries. Following the development of some of these aortic arches, approximately 30 pairs of branches arise from the dorsal aorta, forming the segmental arteries (10–12). Some of the segmental arteries from both sides fuse in the neck to form the vertebral arteries (11, 12). During development, improper fusion of the intra-thoracic segmental arteries is likely the cause of the communication between the intercostal arteries and the vertebral arteries.

Nisar reported a case of a 76-year-old woman who underwent bronchial artery embolization (BAE) for hemoptysis and developed a cerebral infarction the following day (3). Cerebral vascular embolism is a rare complication of BAE, and embolic cerebral infarction predominantly affects the posterior circulation, suggesting that the embolic microspheres or embolic material may have been transmitted through the vertebral arteries. In this case, similar risks were identified, and we implemented a series of measures during the bronchial artery embolization procedure to effectively prevent the reflux of embolic agents into the vertebral artery: (a) imaging guidance: real-time monitoring of the flow of embolic agent throughout the embolization process allows for timely detection and correction of any inappropriate embolization maneuvers; (b) use of microcatheters: superselective embolization is performed using a microcatheter, which allows for more precise control of the release of the embolic agent and minimizes the impact on the peripheral vasculature; (c) appropriate embolic materials: we recommend the use of embolic material with a diameter greater than 500 μm to reduce the risk of regurgitation followed by entry into the vertebral artery through the collateral circulation. In addition, embolization with miniature spring coils is thought to further reduce the risk of regurgitation; (d) controlled injection speed: the injection speed of the embolic agent was carefully controlled, ensuring that it was slow and steady to prevent rapid or forceful injection that could lead to reflux of the agent. Typically, the vertebral arteries arise from the subclavian arteries, pass through the transverse foramina, and converge at the pontine-cerebellar junction to form the basilar artery, which then divides into the posterior cerebral arteries. The vertebrobasilar system supplies structures such as the cerebellum, brainstem, and occipital lobes through its branches. In the case presented here, during angiography, we also discovered a communication between the bronchial artery and the vertebral artery. A possible explanation for this is that, in the presence of chronic lung diseases such as infections, the caliber of the systemic vasculature supplying the lungs may change, potentially leading to a shunt between the systemic and pulmonary circulations, which in turn could cause communication between the bronchial and vertebral arteries (13).

## Conclusion

A thorough preoperative evaluation of the patient’s hemoptysis, along with a comprehensive assessment of ancillary examination results, is essential for making an informed decision. This case highlights the importance of conducting detailed imaging studies and angiography during BAE. Special attention should be given to identifying potential communications between the bronchial arteries and vertebral arteries, as this can help prevent severe complications.

## Data Availability

The original contributions presented in the study are included in the article/supplementary material, further inquiries can be directed to the corresponding author.
